# Prevalence of psychological symptoms and their correlates among physiotherapy clinical students: A cross-sectional study

**DOI:** 10.4102/sajp.v78i1.1795

**Published:** 2022-11-22

**Authors:** Abdulsalam M. Yakasai, Gordana Dermody, Sonill S. Maharaj, Auwal B. Hassan, Auwal Abdullahi, Jibrin S. Usman, Musa S. Danazumi

**Affiliations:** 1 Department of Physiotherapy, Faculty of Health Sciences, University of KwaZulu-Natal, Durban, South Africa; 2 Department of Nursing, School of Nursing, Midwifery and Paramedicine, University of the Sunshine Coast, Queensland, Australia; 3 Department of Physiotherapy, Faculty of Health Sciences, University of Maiduguri, Maiduguri, Nigeria; 4 Department of Physiotherapy, Faculty of Allied Health Sciences, Bayero University, Kano, Nigeria; 5 Discipline of Physiotherapy, School of Allied Health, Human Services and Sport, College of Sciences, Health and Engineering, La Trobe University, Bundoora, Melbourne, Australia

**Keywords:** prevalence, psychological symptoms, depression, anxiety, stress, physiotherapy students

## Abstract

**Background:**

Mental health in medical students is well researched, with physiotherapy students receiving less attention even though psychiatry is a recognised specialty within physiotherapy.

**Objectives:**

To assess the prevalence and correlates of depression, anxiety and stress among physiotherapy clinical students.

**Methods:**

A descriptive cross-sectional study design was employed on 402 physiotherapy clinical students aged 17–40 years using a convenience sampling method. The data were collected using the self-administered 42-items Depression, Anxiety and Stress scale (DASS) and the data were analysed using a Chi-square test and binary logistic regression analysis.

**Results:**

The prevalence of depression, anxiety and stress among these students was 79.9%, 85.6% and 81.6%, respectively. The results indicate that financial status and social life influenced depression by 20.6% (odds ratio [OR] [95%] = 1.206 [1.110, 1.311]) and 36% (OR [95%] = 1.360 [1.050, 1.764]), respectively. Fear of repeating a class influenced anxiety by eight times (OR [95%] = 8.330 [6.643, 10.422]). Fear of repeating a class, financial status and academic performance influenced stress by eight times (OR [95%] = 8.360 [6.677, 10.470]), 17.5% (OR [95%] = 1.175 [1.083, 1.275]) and 18.1% (OR [95%] = 1.181 [1.083, 1.276]), respectively.

**Conclusion:**

Our study concluded that there was a high prevalence of depression, anxiety and stress among physiotherapy clinical students. These outcomes suggest that more attention needs to be given to improving physiotherapy clinical students’ mental health, which will ultimately improve learning outcomes.

**Clinical implications:**

The long-term effects of psychological distress could potentially impact students’ academic performance. It may also have long-lasting effects after graduation. Therefore, students who are at risk of developing psychological symptoms are needed to be thoroughly examined and then receive early required interventions.

## Introduction

Depression, anxiety and stress are major problems among health professions students as they may negatively influence their academic performance and psychological well-being (Janse van Vuuren, Bodenstein & Nel 2018). The psychological and psychosocial changes that students experience during their education are connected to the academic expectations from teachers and family and the development of an autonomous personal life, away from the usual family-centred support (Gjerde 2004). The authors suggest that the current educational system may have inadvertent negative effects on students’ mental health, with a high frequency of depression, anxiety and stress being reported among health professions students (Aktekin et al. 2001; Ball & Bax 2002; Moffat et al. 2004; Raj et al. 2000). Several factors such as voluminous courses, financial constraints, lack of sleep, exposure to clinical activities such as patients’ suffering or deaths and a ‘hidden curriculum’ are believed to contribute to students’ poor mental health (Aktekin et al. 2001; Ball & Bax 2002; Moffat et al. 2004; Raj et al. 2000).

The term hidden curriculum has been discussed in the literature as an unspoken or implicit curriculum that expresses and represents attitudes, knowledge and behaviours, which are conveyed or communicated indirectly by words and actions in the educational setting without aware intent (Alsubaie 2015). While such expectations are not explicitly written, a hidden curriculum in Nigeria may include an unstated promotion or enforcement of certain behavioural patterns, professional standards and social beliefs while navigating a learning environment (Woodhouse & Enukoha 1986).

Studies suggest that psychological distress is prevalent in university students who describe feelings of insomnia, confusion (difficulties in concentration and organisation), loneliness (not living with family) and poor academic performance (Ali et al. 2014; Asante & Andoh-Arthur 2015; Bassi, Sharma & Kaur 2014; Brenneisen et al. 2016; Dyrbye, Thomas & Shanafelt 2006; Hope & Henderson 2014), shortness of breath or eating disorders and financial insecurity (Aktekin et al. 2001; Ball & Bax 2002; Raj et al. 2000). Psychological distress among students may adversely influence their academic performance, contribute to academic dishonesty and play a significant role in alcohol and substance abuse (Ball & Bax 2002; Canadian-Univerities.net 2016; MacLeod et al. 2003; Raj et al. 2000;Stewart et al. 1999; Wear 2002). It has been reported that students who experience psychological distress may become cynical, have deceased empathy and become reluctant to care for the chronically ill (Davis et al. 2001; Griffith & Wilson 2003; Hojat et al. 2004; Wear 2002; Woloschuk, Harasym & Temple 2004). Psychological distress can have long lingering effects after graduation, which could inhibit the new graduates from practicing (Davis et al. 2001; Griffith & Wilson 2003; Hojat et al. 2004; Wear 2002; Woloschuk et al. 2004).

While studies including systematic reviews (Dyrbye et al. 2006; Hope & Henderson 2014) have examined the prevalence of mental health symptoms among health profession students, these studies were largely conducted among medical students with very few studies conducted among physiotherapy students (Abiola, Lawal & Habib 2015; Syed, Ali & Khan 2018). In addition, most of these studies (Dyrbye et al. 2006; Hope & Henderson 2014) were also conducted in other parts of the world where the system of education is well developed compared with sub-Saharan Africa where the education sector is relatively underfunded. Our study aimed to examine the frequency and correlates of psychological symptoms (depression, anxiety and stress) among physiotherapy clinical students attending universities in Nigeria.

## Method

A descriptive cross-sectional study included undergraduate clinical physiotherapy students recruited from eight universities located in different provinces in Nigeria, namely: (1) The University of Nigeria, (2) Bayero University, Kano, (3) University of Ibadan, (4) Obafemi Awolowo University, (5) University of Maiduguri, (6) Nnamdi Azikwe University, Awka, (7) University of Lagos and (8) University of Calabar.

The Nigerian universities are regulated by the National University Commission (NUC) of Nigeria and offer courses and degrees approved by the NUC. All education is provided exclusively in English. The physiotherapy course is spread over 8 months (4 months per semester) with students taking up 9–12 courses per semester. The physiotherapy curriculum is divided into two parts: clinical posting at the university teaching hospitals and classroom lectures. The clinical posting usually begins at 8 am and ends at 1 pm while the classroom lectures start at 2 pm and end at 6 pm. During the clinical posting students are taken to ward rounds through four major physiotherapy units: (1) orthopaedics and surgery, (2) medicine and neurology, (3) paediatrics and obstetrics and (4) gynaecology, where they can demonstrate what they learn from classroom teaching. During classroom lectures, students are taught their registered courses by academics; students are encouraged to ask questions in class and to interact with their peers as applicable. Students are graded based on end-of-semester exams.

Physiotherapy clinical students who were in the 400 level (second to final year) and 500 level (final year) of their undergraduate studies were recruited. Participants were evaluated using a self-reported questionnaire that was used to assess their eligibility. The inclusion criteria were: (1) studying as a full-time student, (2) not currently or previously diagnosed with any psychiatric disorders or eating disorders, (3) not having a cognitive impairment and (4) ability to provide written informed consent. Participants who indicated that they abused drugs and/or alcohol or who failed to turn in their eligibility questionnaire responses were excluded.

### Sample size

The sample size of the study was calculated (a priori) using a formula for cross-sectional studies: *n* = *Z*^2^ *P* (1−*P*) /*d*^2^ where *n* = minimum sample size, Z_α/2_ was set at 0.05 alpha level = 1.96, *P* = based on estimates from a previous study (Abiola et al. 2015) to be 42.6%, and *d* = absolute error or precision (5%). A total of 10% of the entire sample size was used to adjust the sample to give room for non-response (*nr*/*r*−1); therefore, a sample size of 417 individuals was required for our study.

### Ethical considerations

This study was approved by the physiotherapy departments and Health Research Ethics Committees of each university. University students were fully informed in writing of the study purposes and approach and if they were interested in participating, a written informed consent form was signed and submitted.

### Measures

#### Depression, Anxiety and Stress Scale

The 42-item Depression, Anxiety and Stress Scale (DASS) was used to measure the three main psychological domains: depression, anxiety and stress. The DASS is a self-administered instrument that has been validated and used in studies of health profession students (Ali et al. 2014; Dyrbye et al. 2006; Hope & Henderson 2014) and physiotherapy students (Malani et al. 2020; Syed et al. 2018). The depression scale (14 items) assesses dysphoria, hopelessness, devaluation of life, self-deprecation, lack of interest or involvement, anhedonia and inertia (Syed et al. 2018). The anxiety scale (14 items) assesses autonomic arousal, skeletal muscle effects, situational anxiety and subjective experience of anxious effects (Abiola et al. 2015). The stress (14 items) scale is sensitive to levels of chronic non-specific arousal and assesses difficulty relaxing, nervous arousal, being easily agitated, irritability or over-reaction and impatience (Psychology Foundation of Australia n.d.). The scoring and grading of the DASS variables were as follows: depression range 0–9 as normal, 10–13 as mild, 14–20 as moderate, 21–27 as severe, 28+ as extremely severe; anxiety range 0–7 as normal, 8–9 as mild, 10–14 as moderate, 15–19 as severe, 20+ as extremely severe; stress range 0–14 as normal 15–18 as mild, 19–25 as moderate, 26–33 as severe, 34+ as extremely severe (Ali et al. 2014; Syed et al. 2018). The internal consistency reliability (Cronbach’s alpha) for the entire DASS 42 is 0.95. The reliability coefficient for the depression subscale is 0.89; the anxiety subscale is 0.85 and for stress, the subscale is 0.86 (Adetunji & Ademuyiwa 2019).

### Demographics

The demographic information was obtained using a standardised form to allow proper presentation and clarity of data. Each participant was asked to state his or her age, gender, marital status and year of study. The participants were asked to comment about their lecture schedules (bearable or unbearable), mode of living (living with parents, students or alone) and smoking and/or drinking habits (frequently – everyday or most days of the week, occasionally – once in a while and not smoking at all). Their social life was rated by asking the participants to state the number of days they spent doing enjoyable things with others (high – most days of the week, moderate – few days of the week and low – once in a week or occasionally). Fear of failing a class was assessed by asking the participants to rate their fear in percentages (high: ≥ 70%, moderate: ≥ 50% and low ≤ 50%). Academic performance was rated based on the previous academic session’s results (high – indicates results at distinction level: Cumulative Grade Point Average (CGPA) = 4.50–5.00, moderate – indicates results at credit level: CGPA = 3.50–4.49 and low – indicates results at merit level: CGPA = 2.40–3.49). The financial status was assessed based on the Nigerian old minimum wage – 18 000 Naira (high – indicates monthly stipend was above 18 000 Naira, moderate – indicates monthly stipend was around 18 000 Naira and low – indicates monthly stipend was below 18 000 Naira).

### Procedures

Participants were informed of our study via their respective heads of departments and the dean of the faculties and sought their consent using the informed consent link. Participants who provided their consent were invited to complete the questionnaires via email link and WhatsApp message using a Google-drive form and all responses were collected in electronic forms. Our study was conducted in the pre-coronavirus disease 2019 (COVID-19) era from September 2019 to January 2020 and a convenience sampling method was used to collect the data throughout our study period. Reminders were sent to the participants monthly throughout our study period.

### Statistical analysis

Descriptive statistics of frequencies and percentages were used to summarise the demographic information of the participants while the average psychological symptoms (depression, anxiety and stress) were calculated using means and standard deviations. The points of the prevalence of psychological symptoms among the physiotherapy clinical students were computed using percentages. The differences in psychological symptoms between male and female students were analysed using an independent *t*-test after normality has been tested using a Shapiro–Wilk test. The exact Fisher’s Chi-square test was used to compare the prevalence of psychological symptoms between level 400 and 500 students. Shapiro–Wilk test was used to assess the normality of the data and thereafter, Pearson’s product moment correlation (PPMC) was used to determine the relationship between the psychological symptoms and the predictor variables. A multicollinearity check was performed to examine the correlation among the predictor variables and thereafter, a binary logistic regression analysis was conducted to determine the predictability of the variables. The sequential method of entering variables was used to assess the contribution of variables in the models. Model improvement with the addition of each predictor variable, percentage accuracy of each model, *p*-value, standard error and odds ratios (OR) was used in assessing the models (Kim 2017). In addition, model fitness to the data was obtained in the form of the Nagalkerke *R* square. The model with the highest overall percentage correctness that was said to be reliable and at the same time fit the data was then chosen (Kim 2017). Statistical analysis was set at a 5% probability level (*p* < 0.05) and a 95% confidence interval (CI). All data were analysed using SPSS version 23.0 software (SPSS Inc., Chicago, Illinois, USA).

## Results

A total of 417 clinical physiotherapy students from eight universities gave their consent to participate in our study. There were no missing data; however, 15 questionnaires were not returned and therefore, 402 participants returned the questionnaires. This indicates that there was a 3.6% non-response rate. The demographic parameters and points of the prevalence of psychological symptoms among the participants are presented in Table 1. The results indicate that 71.6% (*n* = 288) were males, 28.4% (114) were females, 27.6% (*n* = 111) were between the age of 17–19 years and 72.4% (*n* = 291) were between 20–40 years (Table 1). The clinical parameters of the participants are presented in Table 2.

**TABLE 1:** Demographic characteristics of the participants (*n* = 402).

Variables	*n*	%
**Age**		
17–19	111	27.6
20–40	291	72.4
**Gender**		
Male	288	71.6
Female	114	28.4
**Marital status**		
Married	91	22.6
Not married	311	77.4
**Academic level**		
400	207	51.5
500	195	48.5
**Academic performance**		
High	83	20.6
Moderate	211	52.5
Low	108	26.9
**Financial status**		
High	117	29.1
Moderate	201	50.0
Low	84	20.9
**Fear of failing a class**		
High	277	68.9
Moderate	63	15.7
Low	62	15.4
**Lecture schedules**		
Bearable	312	77.6
Unbearable	90	22.4
**Mode of living**		
Living with parents	169	42.0
Living with students	135	33.6
Living alone	98	24.4
**Social life**		
High	123	30.6
Moderate	176	43.8
Low	103	25.6
**Smoking/drinking**		
Frequently	7	1.74
Occasionally	46	11.44
Not at all	349	86.8

**TABLE 2:** Clinical parameters of the participants (*n* = 402).

Variables	*n*	%
**Depression**		
Normal	81	20.1
Mild	63	15.7
Moderate	166	41.3
Severe	51	12.7
Extremely severe	41	10.2
Mean ± standard deviation	13.02 ± 2.31	-
Point prevalence	321	79.9
**Anxiety**		
Normal	58	14.4
Mild	76	18.9
Moderate	156	38.8
Severe	76	18.9
Extremely severe	36	9.0
Mean ± standard deviation	17.49 ± 3.67	-
Point prevalence	344	85.6
**Stress**		
Normal	74	18.4
Mild	102	25.4
Moderate	173	43.0
Severe	30	7.5
Extremely severe	23	5.7
Mean ± standard deviation	21.04 ± 5.33	-
Point prevalence	328	81.6

Note: Normal (0–9 for depression, 0–7 for anxiety and 0–14 for stress); mild (10–13 for depression, 8–9 for anxiety and 15–18 for stress); moderate (14–20 for depression, 10–14 for anxiety and 19–25 for stress); severe (21–27 for depression, 15–19 for anxiety and 26–33 for stress); extremely severe (28+ for depression, 20+ for anxiety and 34+ for stress).

The overall result (for both levels 400 and 500) indicates that the average level of depression was mild (13.02 ± 2.31), the average level of anxiety was severe (17.49 ± 3.67) and the average level of stress was moderate (21.04 ± 5.33) among the students. In addition, the point prevalence of depression, anxiety and stress was 79.9%, 85.6% and 81.6% among the students (Table 2).

The difference in psychological symptoms between male and female students is presented in Table 3. The results show that female students have higher levels of depression (female vs. male: 15.61 [3.21] vs. 10.43 [4.04], *p* = 0.003) and stress (female vs. male: 23.63 [4.88] vs. 18.45 [5.03], *p* = 0.021) compared with male students; however, there was no significant difference in anxiety levels (female vs. male: 18.03 [2.96] vs. 16.95 [3.11], *p* = 0.062) between the students. The difference in the point prevalence of the psychological symptoms between level 400 and 500 students is presented in Table 4.

**TABLE 3:** Difference in psychological symptoms between male and female students.

Variables	Male (288)		Female (114)		Mean difference	95% CI	*p*
	Mean	s.d.	Mean	s.d.			
Depression	10.43	4.04	15.61	3.21	−5.17	−5.27, −5.07	0.003*
Anxiety	16.95	3.11	18.03	2.96	−1.08	−1.09, −1.07	0.172
Stress	18.45	5.03	23.63	4.88	−5.18	−5.28, −5.07	0.012*

s.d., standard deviation; CI, confidence interval.

* , Significance.

**TABLE 4:** Chi-square test of difference in psychological symptoms between level 400 and 500 students.

Variables	Percent	95% CI	*X* ^2^	*df*	*n*	*p*
**Depression**			164.6	1	402	0.03*
400 students	54.4	44.0, 64.8				
500 students	79.7	69.5, 89.9				
**Anxiety**			231.7	1	402	0.01*
400 students	68.9	58.3, 79.5				
500 students	46.7	57.5, 80.3				
**Stress**			136.4	1	402	−0.04*
400 students	58.1	47.7, 68.5				
500 students	46.0	33.7, 58.3				

CI, confidence interval; *X*^2^, chi-square; *df*, degree of freedom; *N*, sample size.

* , Significance.

The results indicate that there were significant differences between level 400 and 500 students in depression (level 400 vs. 500: 54.4% [44.0, 64.8] vs. 79.7% [69.5, 89.9], *p* = 0.03), anxiety (level 400 vs. 500: 68.9% [58.3, 79.5] vs. 46.7% [57.5, 80.3], *p* = 0.01) and stress (level 400 vs. 500: 58.1% [47.7, 68.5] vs. 46.0% [33.7, 58.3], *p* = 0.04), with level 500 students having higher depression (79.7%) and level 400 having higher anxiety (68.9%) and stress (58.1%) (Table 4).

**FIGURE 1:**
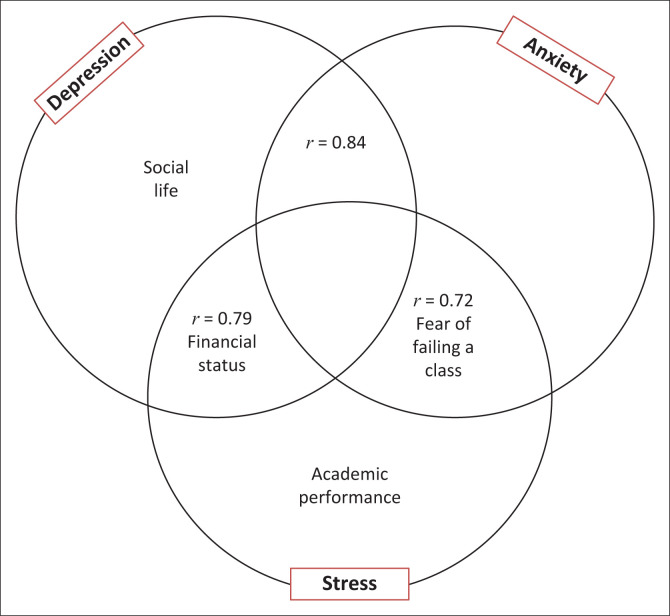
A cascade effect among the psychological symptoms and the predictor variables.

The relationship between the psychological symptoms and the predictor variables is presented in Table 5. The result shows a cascade effect with depression, anxiety and stress significantly correlated with each other. The result also indicates that two variables (financial status and social life) were correlated with depression, one variable (fear of failing a class) was correlated with anxiety and three variables (financial status, fear of failing a class and academic performance) were correlated with stress (Figure 1 and Table 5). The influence of the predictor variables on psychological distress is presented in Table 6. The results indicate that financial status and social life influence depression by 20.6% (OR [95% CI] = 1.206 [1.110, 1.311]) and 36% (OR [95% CI] = 1.360 [1.050, 1.764]), respectively. Fear of failing a class influenced anxiety by eight times or 733% (OR [95% CI] = 8.330 [6.643, 10.422]). Fear of failing a class, financial status and academic performance influenced stress by eight times or 736% (OR [95% CI] = 8.360 [6.677, 10.470]), 17.5% (OR [95% CI] = 1.175 [1.083, 1.275]) and 18.1% (OR [95% CI] = 1.181 [1.083, 1.276]), respectively (Table 6).

**TABLE 5:** Correlation among psychological symptoms and predictor variables.

Variables	Psychological distress					
	Depression		Anxiety		Stress	
	*r*	*p*	*r*	*p*	*R*	*p*
Depression	-	-	0.84***	0.001	0.79***	0.001
Stress	-	-	0.72***	0.001	-	-
Age	0.04	0.47	0.01	0.67	0.06	0.67
Gender	0.03	0.62	0.15	0.75	0.11	0.31
MRS	0.13	0.32	0.07	0.28	0.08	0.46
ACL	0.12	0.58	0.01	0.40	0.13	0.52
ACP	0.11	0.60	0.09	0.58	0.51*	0.02
FNS	0.57**	0.03	0.06	0.86	0.65*	0.03
FRC	0.08	0.78	0.71**	0.01	0.74**	0.01
LCS	0.02	0.52	0.04	0.83	0.02	0.51
MOL	0.05	0.49	0.03	0.45	0.01	0.32
SLF	0.53*	0.04	0.21	0.32	0.14	0.37
S/D	0.02	0.97	0.01	0.73	0.03	0.29

*r*, Coefficient of correlation; MRS, marital status; ACL, academic level; ACP, academic performance; FNS, financial status; FRC, fear of repeating class; LCS, tight lecture schedules; MOL, mode of living; SLF, social life; S/D, smoking/drinking.

* , Correlation is significant at the 0.05 level (2-tailed).

** , Correlation is significant at the 0.01 level (2-tailed).

*** , Correlation is significant at the 0.001 level (2-tailed).

**TABLE 6:** Influence of the predictor variables on the psychological distress.

Variables	B	s.e.	OR	95% CI	*p*	Nagalkerke *R*^2^
**Depression**						0.681
Financial status	0.188	0.043	1.206	1.110, 1.311	0.021*	
Social life	0.310	0.132	1.360	1.050, 1.764	0.033*	
**Anxiety**						0.734
Fear of failing a class	2.120	0.114	8.330	6.643, 10.422	0.001*	
**Stress**						0.764
Fear of failing a class	2.122	0.120	8.360	6.677, 10.470	0.001*	
Financial status	0.162	0.042	1.175	1.083, 1.275	0.043	
Academic performance	0.162	0.042	1.181	1.083, 1.276	0.015*	

B, Beta; s.e., standard error; CI, confidence interval; OR, odds ratio.

* , Significance.

## Discussion

Our study was conducted to examine the prevalence and correlates of depression, anxiety and stress among physiotherapy clinical students. The findings indicate that the point prevalence of depression, anxiety and stress among the students were 79.9%, 85.6%, and 81.6%, respectively. These findings are similar to those of Malani et al. (2020) who reported that the prevalence of psychological symptoms among physiotherapy students as high (depression – 53%, anxiety – 64% and stress – 65%). Similar findings were also reported that indicated that the prevalence of depression was high among university students (Emmanuel 2016; Fushimi, Saito & Shimizu 2013; Ibrahim et al. 2013).

Female students have higher levels of depression and stress compared with male students; however, there was no significant difference in anxiety levels between the students. These findings are similar to those of Almhdawi et al. (2018) who studied the prevalence of mental health symptoms among 838 allied health professional students. Their findings indicated female students as having higher levels of stress and depression than male students. Several factors including abuse, education and income have been linked to the increase in depression and stress among females; however, published evidence suggests that biological factors, such as ovarian hormone levels and particularly decreases in oestrogen may contribute to the increased prevalence of depression and anxiety in women (Emmanuel 2016). On the other hand, the presence of androgen receptors in men may confer protection, for example, in hippocampal neurons, which become reduced with depression (Kim 2017). In addition, as testosterone does not cycle in men as oestrogen does in women, there may be more consistent protection in men (Ibrahim et al. 2013). However, men also have sexually dimorphic brain nuclei, particularly in the hypothalamus, so the lower prevalence of depression in men is probably more complex owing not only to hormonal differences but also to developmental differences in brain circuitry (Syed et al. 2018).

There were statistically significant differences in psychological symptoms between level 400 and 500 students with students from 400 levels having fewer depression symptoms than 500 level students; however, level 400 students reported higher anxiety (68.9%) and stress (58.1) symptoms than level 500 students. The higher symptoms of anxiety and stress found among level 400 students may be attributed to their first clinical experience and high study pressure possibly stemming from large-volume classwork. Moutinho et al. (2017) investigated the prevalence of psychological symptoms among Brazilian medical students and the results showed that these symptoms were endemic among medical students possibly because of the nature and workload of the medical course, as well as because of the academic structure of the course and its teaching methods. Our findings are supported by Shaikh and colleagues (2004) in Pakistan and Saipanish (2003) in Thailand who reported a higher level of stress and anxiety among third- and fourth-year medical students. The reasons for such a high level of stress among fourth-year students were attributed to both environmental (nature of the clinics, hospitalisation and death) and personal factors (fear of failing a class, financial status and drug abuse) (Shaikh et al. 2004). In addition, during the last university years, there are quite significant changes in students’ lifestyle, such as writing a thesis, preparing for exams and worrying about securing a job after graduation (Shaikh et al. 2004). These factors could exacerbate stress and anxiety and may subsequently increase depression symptoms among students (Rees et al. 2009) and may also explain the reason why level 500 students showed higher depression symptoms than level 400 students.

We showed a cascade effect with inter-relationships among depression, anxiety and stress. The results also indicate that financial status and social life correlated with depression, fear of failing a class correlated with anxiety and financial status and fear of failing a class and academic performance correlated with stress. This could be because of a lack of integration of financial, academic and social support. There is also a lack of engaging students with support programmes possibly because of insufficient data about students identifying their own support needs as well as limited adaptation of programmes to meet the specific expectations of students. This was supported by Janse van Vuuren et al. (2018) who indicated a lack of integration of academic and emotional support among physiotherapy students in South Africa.

In contrast, no statistically significant relationships were found between other variables (age, gender, marital status, academic level, tight lecture schedules, mode of living and smoking and/or drinking) and psychological symptoms (depression, anxiety and stress). Our findings are similar to those of Jadoon et al. (2010) who found that age and marital status did not significantly correlate with psychological symptoms among 482 Pakistani medical students. Contrary to our findings, Jadoon et al. (2010) reported that students’ academic levels significantly correlated with depression and anxiety.

Financial status and social life were the strongest predictors of depression. This finding is similar to what Peltzer et al. (2013) found among 820 randomly selected Nigerian students. Our findings on stress and anxiety levels correlated with academic performance and fear of failing a class. Contrary to our findings, Yadav, Gupta and Malhotra (2016) found that family problems, substance abuse and staying in hotels were the significant predictors of depression and anxiety among 330 Indian medical students. Yusoff et al. (2013) reported that desire and derive-related stresses were associated with stress, anxiety and depression among 762 Malaysian medical students. These significant differences suggest that the correlates of psychological symptoms among health professions students are diverse with significant variations existing among cultures and settings.

### Clinical relevance

The effects of psychological distress could impact students’ academic performance while at university. It may also have long-lasting effects after graduation and ultimately impact students’ practice and patients’ care. As professionals we know that distress does not go away after graduation, students who are at risk of developing psychological symptoms are needed to be thoroughly examined and then attended to by the relevant healthcare professionals where applicable.

### Limitations

The cross-sectional nature of our study made it difficult to establish cause and effect; therefore, the findings must be interpreted with caution. However, a cross-sectional study has the advantage of being able to investigate a large group of people at a single point in time and thus may be used to study the prevalence, identify correlates and predict associations. In addition, our findings also relied on self-reported data, which could have been subjected to reporting bias. Furthermore, the instrument used cannot be used to diagnose psychological symptoms but can be used to screen for their presence.

A follow-up study with focus group discussion could be carried out to get a deeper understanding of students’ psychological symptoms, implement interventions and assess their effects. The study population could also be expanded to identify psychological symptoms of pre-clinical physiotherapy students to determine the relevance and efficacy of the proposed student support structure.

## Conclusion

Our findings indicated that there was a high prevalence of depression, anxiety and stress among physiotherapy clinical students in eight Nigerian universities. It is recommended that universities and governments should develop programmes to support learning and teaching methods that could address the high prevalence of depression, anxiety and stress among physiotherapy students and enhance students’ well-being and learning success.
